# Inorganic Crystal Structure Database (ICSD) and Standardized Data and Crystal Chemical Characterization of Inorganic Structure Types (TYPIX)—Two Tools for Inorganic Chemists and Crystallographers

**DOI:** 10.6028/jres.101.022

**Published:** 1996

**Authors:** Ekkehard Fluck

**Affiliations:** Gmelin-Institut for Inorganic Chemistry of the Max Planck Society, D-60486 Frankfurt, Germany

**Keywords:** compare, ICSD, inorganic structure data bases, LAZY PULVERIX, RETRIEVE, STRUCTURE TIDY, TYPIX

## Abstract

The two databases ICSD and TYPIX are described. ICSD is a comprehensive compilation of crystal structure data of inorganic compounds (about 39 000 entries). TYPIX contains 3600 critically evaluated data sets representative of structure types formed by inorganic compounds.

## 1. Introduction

A systematic search for new materials requires a firm knowledge of crystal structures and the building principles of condensed matter. The knowledge of crystallographic data is also the basis for many theoretical considerations. The amount of available crystallographic data is continually increasing. By 1995 some 40 000 inorganic structures had been described, ignoring oxides and halides. In this paper I would like to present two databases which are compilations of crystal structure data of inorganic compounds, the Inorganic Crystal Structure Database^1^ marketed under the acronym ICSD and a smaller database of inorganic structure types called TYPIX.

## 2. Inorganic Crystal Structure Database (ICSD)

The Inorganic Crystal Structure Database (ICSD) was initiated by Professor Bergerhoff in 1978 at the Institute of Inorganic Chemistry of the University of Bonn. Since 1989 ICSD has become a joint venture of the Gmelin Institute and FIZ Karlsruhe. ICSD is a comprehensive compilation of crystal structure data of inorganic compounds. It contains information on compounds which

- have no C–C and/or no C–H bonds in any of their residues
- and which include at least one of the following nonmetallic elements: H, He, B, C, N, O, F, Ne, Si, P, S, Cl, Ar, Se, Br, Kr, Te, I, Xe, At, and Rn.

All information stored in ICSD is taken from the primary papers on the subject and to a small extent from private communications. The literature is covered back to 1915. The current version of the database contains about 39 000 entries and is updated with approximately 1800 new entries per year. Since 1993 the CD-ROM version of the ICSD database can be accessed with the retrieval software RETRIEVE, an interface that provides pull-down menus, dialog boxes, and mouse support.

In most cases the user will start his search with the chemical composition. From the periodic table he can select

- single elements with oxidation state and stoichiometric index
- main or sub groups
- number of different elements occurring in a compound
- and finally the formula type (for example A_2_BX_4_)

The menu allows a search for the following:

crystal system
Bravais lattice
center of symmetry
space group symbol
space group number
crystal class
Pearson symbol
Laue class symbol

For a typical ICSD entry the screen displays the chemical name and formula, mineral name if it is a mineral, title of the paper and reference, author’s name, crystal data, cell volume, formula units, oxidation states of the atoms, etc. A calculation menu allows one to calculate interatomic distances. From an option menu the user can select the type of data to be displayed on the screen. A crystal visualizer allows the visualization of all ICSD database structures as 3D drawings.

Before coming back to this feature a new version of the retrieval software which was introduced in the 1995 Spring update of the Inorganic Crystal Structure Database may be mentioned. The main innovation is the so-called COMPARE module. It serves to detect similarities between ICSD entries, i.e., it should help to decide whether the entries are isotypical or not. In principle, the program compares the parameter list of two isopointal structures. The compilation of isopointal structures is possible on the basis of another new search routine which collects structures with the same Wyckoff sequence. As it is highly recommended to compare standardized structures only, the COMPARE module includes an automatic standardization routine. The standardization process is based on the theory of Parthé and others, and I shall refer to this program a little bit later. Also, COMPARE calculates a value which represents the average of all differences between coordinate triplets of corresponding atom sites. Symmetry-equivalent atoms in neighboring cells are also considered. The value may serve as an indicator of isotypism. Other new features of the retrieval software include

- the standardization of single ICSD entries with the program STRUCTURE TIDY
- powder pattern simulation of ICSD entries with the program LAZY PULVERIX
- a search module for coordination numbers within defined distance ranges
- an improved sorting module
- and extended export functions, for instance, exporting to CIF format.

The crystal visualizer CVIS mentioned before is a menu-driven program which allows the visualization of all ICSD database structures as 3D drawings. The program can be called up as an application from the RETRIEVE program, but it can be used separately with data from other sources, such as the Cambridge file or a SHELX file. The program CVIS draws molecules and other discrete building units automatically from the input dataset, applying symmetry operators of all settings and using an internal list of atomic distances. It is specifically designed to draw inorganic structures characterized by an infinite lattice. Thus, important parts of the structure can be selected, for instance, a phosphate chain, and can be built up automatically or stepwise, that is sphere by sphere, by simple commands. The program is able to visualize up to 2000 atoms and to draw parallel and perspective projections. The size and color of atoms can be freely chosen. Another feature is the manual or automatic search of bonds. For visualization several models can be chosen such as ball-and-stick, wire, or calotte. The program allows continuous rotation, translation, and enlargement or reduction; interactive measurements of distances, angles, and torsions; and histograms of statistical distribution of distances between selected atoms. Of course, structures can be printed on graphic printers. The broader variation of atomic distances can be taken into consideration by setting upper and lower limits in a histogram of distance distributions of selected atom pairs.

The present version of the ICSD is available

- By leasing the retrieval software for in-house use on a PC or a main frame. It is distributed by
  FIZ Karlsruhe
  D-76344 Eggenstein-Leopoldshafen
  Fed. Rep. of Germany
  Tel.: (+49)7247-808-253
  Fax: (+49)7247-808-666
  In the USA and Canada:
  Scientific Information
  Service Inc.
  7 Woodland Avenue
  Larchmont, NY 10538, USA
  Tel.: (914)834-8864
  Fax: (914)834-8903
  In Japan:
  JAICI
  Nakai Building
  6-25-4 Honkomagome, Bunkyo.ku
  Tokyo 113, Japan
  Tel.: (3) 5978-3622
  Fax: (3) 5978-3600
- By interactive online access via telecommunication connections to STN International
- On CD-ROM through agreement with the International Union of Crystallography (IUCr). This special agreement allows an inexpensive distribution of the CD-ROM version of ICSD to individual units of academic institutions and to individuals at academic institutions. For this CD-ROM version with the software RETRIEVE and VISUALIZER, an IBM-compatible PC with DOS is required. The price of the ICSD CD-ROM for an annual license is DM 1200 for individual units of academic institutions and DM 500 for single individual users at academic institutions. Orders must be placed for a period of 3 years and cannot be cancelled. We hope that this special program will result in a much wider distribution of ICSD in the academic world.

The distribution licenses for commercial and industrial users, networks, and campus licenses as well as for other distribution media, such as online access or distribution on magnetic tape, are not affected by the special academic program.

## 3. Standardized Data and Crystal Chemical Characterization of Inorganic Structure Types (TYPIX)

TYPIX is a critical compilation of crystallographic data for about 3600 compounds, representing structure types found among inorganic compounds. Structure types found exclusively among halides or oxides are included in specific cases only. TYPIX also contains condensed crystal-chemical information about individual structure types and an extensive chapter on the crystal chemistry of particular structure families. The purpose of this compilation is to clarify and classify published data for intermetallic and other inorganic structures. It provides a tool for further crystal-chemical studies and the development of new materials. TYPIX was first published in 4 volumes within the GMELIN Handbook of Inorganic and Organometallic Chemistry. Volume 1 contains a chapter on the standardization of crystal structure data. The standardization procedure which has been developed at the University of Geneva by Professor Parthé and his group has greatly facilitated the identification of distinct structure types by selecting one single data set from a large number of equivalent crystallographic descriptions. The procedure applies unambiguous criteria for the space group setting, the unit cell parameters, the representative triplets, and the origin and rotation of the coordinate system. With the program STRUCTURE TIDY mentioned before, the reader can standardize his own data. Standardized crystallographic data can be used directly for an application requiring a standard setting. Volume 1 of the printed version also contains a chapter on the crystal chemical characterization of inorganic structure types. There are 57 tables, most of them with drawings, which group structure families which are analyzed according to various crystal chemical concepts, such as structures with close-packed layers, structures with intergrown slabs, columns, or blocks; structures with particular coordinations or linkages, and deformation, substitution; and filled-up or vacancy derivatives. Volume 2 contains cross reference tables which order the structure types according to the following:

- colloquial name,
- Pearson code,
- space group,
- chemical formula of the type-defining compound.

Volumes 3 and 4 contain the main data tables for the various structure types, i.e., triclinic, monoclinic, orthorhombic, tetragonal, trigonal, hexagonal, and cubic. They contain complete crystallographic data sets for 3206 structure types, ordered according to the space group, the Pearson code and the Wyckoff sequence. The standardized crystallographic data for the most recent refinement of the type-defining compounds have remarks about corrections to the original data. All crystallographic data for type-defining compounds were checked to determine whether the distance is too short or the symmetry elements have been overlooked. For 296 types misprints in the published crystallographic data were corrected; 116 types were referred to a space group of higher symmetry than the one used in the original publication; for 42 types a data set in a space group of higher symmetry to be tested in future refinement was added, and for 239 types pseudo-symmetry was indicated. There were 186 structure types which have been stated in the literature to be wrong and were excluded from the compilation but are mentioned in remarks. The main data tables also contain remarks about related literature, additional symmetry, reference to related structure types, etc. The STRUCTURE TIDY program is added to Volume 4 on the diskette. An updated and computerized version of Volumes 2 – 4 of TYPIX in the GMELIN Handbook is now available. There are 3600 critically evaluated data sets, representative of structure types formed by inorganic compounds covering the literature up to January 1995, which can now be readily accessed on your PC. The retrieval program allows searches combining tests on bibliographic, crystallographic, and crystal chemical features. The crystallographic data are standardized with respect to the choice of the space group setting, cell parameters, origin and rotation of the coordinate system, and representative triplets. Short geometric descriptions emphasize structural subunits, close-packed layers, coordination polyhedra, etc. There are 2000 cross-references which point to deformation, substitution, and filled-up and intergrowth derivatives. The CD-ROM also contains the programs for standardizing crystallographic data and for calculating powder diffraction diagrams for type-defining and isotypic compounds.

The TYPIX database (MS-DOS version for IBM-PC compatible, processor 386 or higher, 15 MB hard disk space) is available from:

Gmelin-Institute for Inorganic Chemistry,
Varrentrappstr. 40/42
D-60486 Frankfurt/Main, Germany
FAX: +49-69-7917-338

## Footnotes

1 Certain commercial equipment, instruments or materials are identified in this paper to foster understanding. Such identification does not imply recommendation or endorsement by the National Institute of Standards and Technology, nor does it imply that the materials or equipment identified are necessarily the best available for the purpose.

